# Polygenic *in vivo* validation of cancer mutations using transposons

**DOI:** 10.1186/s13059-014-0455-6

**Published:** 2014-09-27

**Authors:** Su Kit Chew, Dong Lu, Lia S Campos, Kenneth L Scott, Abdel Saci, Juexuan Wang, Adam Collinson, Keiran Raine, Jonathan Hinton, Jon W Teague, David Jones, Andrew Menzies, Adam P Butler, John Gamble, Sarah O’Meara, Stuart McLaren, Lynda Chin, Pentao Liu, P Andrew Futreal

**Affiliations:** Wellcome Trust Sanger Institute, Hinxton, UK; Current address: UCL Cancer Institute, London, UK; Current address: Babraham Institute, Cambridge, UK; Baylor College of Medicine, Houston, TX USA; Novartis Institutes for Biomedical Research, Cambridge, MA USA; University of Texas MD Anderson Cancer Center, 1515 Holcombe Blvd, Houston, TX 77030 USA

## Abstract

The *in vivo* validation of cancer mutations and genes identified in cancer genomics is resource-intensive because of the low throughput of animal experiments. We describe a mouse model that allows multiple cancer mutations to be validated in each animal line. Animal lines are generated with multiple candidate cancer mutations using transposons. The candidate cancer genes are tagged and randomly expressed in somatic cells, allowing easy identification of the cancer genes involved in the generated tumours. This system presents a useful, generalised and efficient means for animal validation of cancer genes.

**Electronic supplementary material:**

The online version of this article (doi:10.1186/s13059-014-0455-6) contains supplementary material, which is available to authorized users.

## Background

The sequencing of cancer genomes has become a globally coordinated effort to understand the genomic, epigenetic and gene expression changes that occur in cancers [1]. By coupling the output of the catalogues of mutations and alterations in cancer genomes with systematic functional analyses, an overarching aim is to derive both an understanding of the underlying pathophysiological process and improved clinical outcomes [2-4]. The functional validation and characterisation of candidate cancer mutations presents a practical challenge in cancer genomics, due to the diversity of assays and in particular the costs and duration of *in vivo* experiments.

DNA transposons are mobile genetic elements that translocate within the genome via a ‘cut-and-paste’ mechanism. They are versatile genetic tools used in a variety of genetic models for purposes ranging from transgene delivery to mutagenesis and chromosome engineering [5,6]. The *Sleeping Beauty* (*SB*) transposon is active in mammalian cells and has been used for cancer gene discovery in a variety of tissue contexts by insertional mutagenesis [7-10]. The *piggyBac* (*PB*) transposon is a DNA transposon recently described to be active in mouse ES cells with much higher transposition efficiency than *Sleeping Beauty* [11,12]. We have previously shown that enhancer/transcript-trapping DNA transposons targeted into the genome can be used to assay the *in vivo* oncogenic potential of the payload cDNA contained in the transposon [13]. Tumourigenesis in this system requires the confluence of appropriate genetic, temporal and microenvironment contexts: first, the transposon must carry an oncogenic cDNA; second it traps a transcript that expresses the cDNA at an appropriate level; third, this occurs in a susceptible tissue and at a proper developmental stage; and finally, the cDNA confers a selective advantage so that expression is maintained by positive selection. A bacterial artificial chromosome vector constructed to carry an array of transposons with different cDNAs was targeted to the *Hprt* locus using recombination mediated cassette exchange in ES cells. When animals generated from these ES cells were crossed to a strain ubiquitously expressing transposase, the progeny developed a broad spectrum of tumours that expressed the oncogenic cDNAs but not the control cDNAs from the transposon array. The transposons stochastically mobilise in somatic cells, expressing their payload cDNA when they insert near enhancers or expressed genes and trap the expression activity. Due to the ubiquitous expression of transposase, transposons can also remobilise and lose payload cDNA expression. Therefore, the expression of an individual or group of transposon cDNA(s) clonally maintained within an expanding tumour over time would suggest positive clonal selection for those cDNA(s). This approach effectively assays the oncogenic potential of each cDNA payload in multiple tissue contexts. As transposition is somatic and cell-autonomous, each somatic cell would have a unique transposon insertion profile and a single animal line with a library of transposons could develop tumours driven by different combinations of oncogenic cDNAs.

To practically use transposon-mediated *in vivo* tumourigenesis to functionally validate cancer mutations, we required an efficient method for transfecting transposons into the murine genome and a strategy for tracking the transposons. To address those needs, we describe here a nested PB-SB transposon vector that allows pools of multiple transposons to efficiently transduce into the genome of mouse embryonic stem (ES) cells [12]. Individual transposons constructs are tagged with unique 3’ UTR sequences to track the exogenous cDNAs and facilitate genotyping. We used this system to assay the *in vivo* oncogenic potential of a set of kinase mutations we have previously observed in human cancers [14], recovering tumours that recurrently expressed a subset of the cDNAs. The tumours show selective retention of certain cDNAs during serial transplantations and generation cell lines. Exome sequencing of the tumours revealed intratumour heterogeneity and evolutionary life histories similar to that observed in human cancers. In two examples of mutations in *Cyclin-dependent kinase 2* (*CDK2*) and *Diacylglycerol kinase, beta* (*DGKB*) genes, we show how the *in vivo* assay reveals novel insights on the functions of these genes in cancer. This system uses standard molecular biology and transgenic protocols, yielding a general mouse model for validating cancer mutations in a wild-type background that is polygenic in throughput. This *in vivo* validation system complements and informs *in vitro* cell-based assays in functional cancer genomics.

## Results and discussion

### Design and generation of transposon constructs

**Figure 1:**
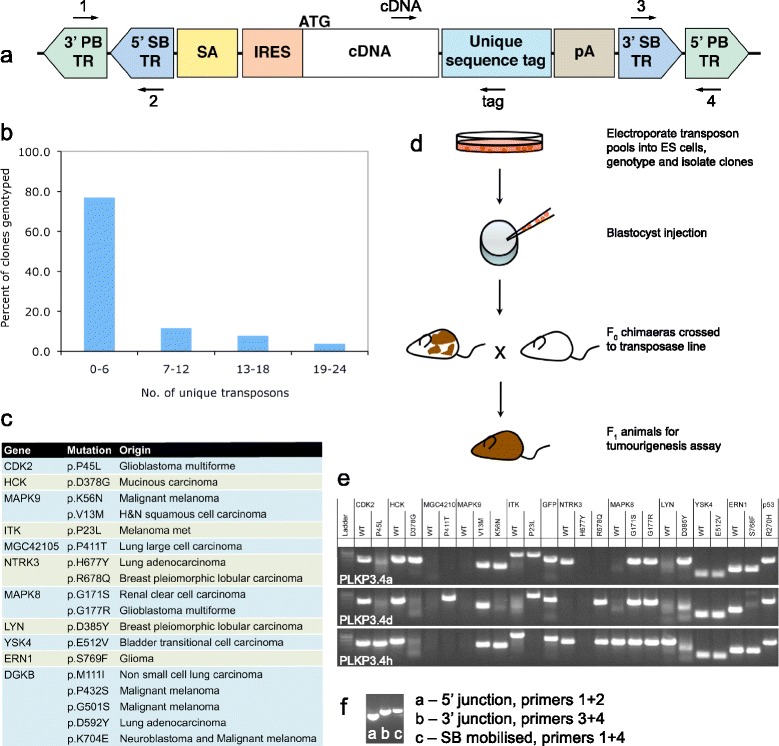
**Transposon mediated** ***in vivo*** **validation of cancer mutations. (a)** Schematic for design of transposon construct, not to scale. Arrows show relative primer positions for subsequent genotyping and PCR assays. ATG, translation start codon of incorporated cDNA; IRES, encephalomyocarditis virus internal ribosome entry site; pA, bovine growth hormone poly-adenylation signal; PB, piggyBac; SA, *Engrailed* splice acceptor; SB, Sleeping Beauty; TR, terminal repeat. **(b)** Distribution of unique transposon species in pooled electroporation. A pool of 24 unique sequence-tagged transposons containing the neomycin resistance marker is electroporated into murine ES cells and colonies selected for in media supplemented with geneticin. Distribution shown is for 73 picked clones that are geneticin-resistant. **(c)** List of kinase mutations and the tumour type where they were observed in human patients. For each kinase both the wild-type and mutant versions of the cDNA were constructed and incorporated into individually tagged transposon constructs. **(d)** Schematic of the experimental strategy for *in vivo* validation of candidate cancer gene alleles using transposon. **(e)** Genotyping PCR with forward primer in cDNA and reverse primer in sequence tag (see panel a) to detect presence of individual transposons. Each row shows an individual animal with the 25 PCR reactions to the different transposons, 3 F1 progeny from a litter are shown. **(f)** In F1 pups with SB transposase, both intact transposon (detected by the junction primers) and SB mobilisation (detected by the flanking primers) are detectable (primers shown in Figure 1a). Loss of the nested SB transposon results in the PCR reaction amplifying a product of similar size to the terminal repeat junctions.

To transfect pools of transposons into the genome of cells without constructing or manipulating large arrays of transposons in bacterial artificial chromosome constructs that were required previously [13], we rationalised that we could take advantage of the high transposition efficiency of *piggyBac* that is two to three orders of magnitude higher than the *Sleeping Beauty* transposon in ES cells [12]. By nesting the rest of the transposon vector design within *PB* terminal repeats (Figure 1a), we can efficiently introduce pools of cargo payload within the *PB* repeats into the genome of cells by co-electroporating transposon constructs with a helper plasmid encoding *PB* transposase. The nested *PB* and *SB* transposon design (Figure 1a) permits us to use either *PB* or *SB* transposase to mobilise the transposon *in vivo*. As our intention was to assay the oncogenic potential of the cDNA payload itself and not the oncogenic potential from disruption of an endogenous loci or novel fusion transcripts, we introduced an internal ribosomal entry sequence (IRES) after the splice acceptor sequence so that the cDNA payload is expressed alone. We incorporated a 60-basepair unique sequence tag between the stop codon and poly-A signal (Figure 1a and see Additional file 1: Table S1). This tag allows us to specifically detect the presence of the transposon in the genome and expression of the cDNA transcript by PCR, and eliminates possible non-specific amplification from the endogenous gene.

To evaluate the efficiency of the pooled electroporation strategy, we combined a pool of 24 unique sequence-tagged *PB* transposons constructs with the transposase helper plasmid for electroporation into mouse ES cells. Co-electroportation led to a genomic distribution of unique transposon insertions where approximately 20% of 73 genotyped Neomycin-resistant clones have greater than six unique transposons using PCR genotyping of the sequence tags (Figure 1b). This frequency distribution demonstrates the utility of pooled transposon electroporation and suggests that the probability of each transposon integrating is independent of the other constructs.

For the candidate cDNA payloads, we curated a list of 18 kinase mutations from a variety of cancers [14] (Figure 1c), chosen for their likelihood to function as dominant gain-of-function point mutations based either on the location of the mutation in the functional domains or the distribution of mutations within the gene. In addition to the mutant alleles, we also constructed transposons with the 11 wild-type cDNA sequences to compare the effects of ectopic expression of the kinases.

### Generation of animals for tumourigenesis assay

The schematic illustrating animal generation for *in vivo* tumourigenesis is outlined in Figure 1d. We electroporated pools of six to 25 transposon constructs together with the *piggyBac* transposase helper plasmid into AB2.2 and JM8A3 mouse ES cell lines [15,16]. Different representations of transposons were identified in the electroporated ES cell clones by PCR genotyping using primers specific to each cDNA and its corresponding unique sequence tag (Figure 1a and Additional file 1: Table S1). We used ES cell clones that each carried four to 21 unique transposons to generate chimaera animals by blastocyst microinjection (Additional file 1: Table S2). The pools were combinations of mutations in different genes, and a pool of different mutations in the same gene (DGKB). Potential passenger effects due to insertional mutagenesis by the transposon are avoided by having multiple animal lines generated from different ES cell clones with different pools and initial insertion profiles, so that recurrent expression of oncogenic cDNA transposons recovered from different tumours would have originated from cells with distinct initial transposon integration sites (Additional file 1: Table S2). In parallel, we electroporated transposon pools into ES cell lines already expressing either the *PB* or *SB* transposase, the chimaeras generated from these ES cell clones have mobilised transposons and can be used directly for the tumourigenesis assay in the F0 generation chimaeras. To provide a sensitised background for tumourigenesis, we also co-electroporated a subset of ES cells with a transposon constitutively expressing the dominant-negative R270H allele of the *Trp53* tumour suppressor gene [17] under the control of the CAGG (cytomegalovirus early enhancer and chicken beta-actin promoter hybrid) synthetic promoter.

Chimaera animals with pools of transposons in their genome were subsequently crossed to a mouse line expressing the SB11 transposase from the ubiquitous *Rosa26* promoter [10]. In the F1 progeny of this cross, we are able to detect individual transposons in genomic DNA using primers designed to amplify the cDNA and its unique sequence tag (Figure 1e). While Mendelian segregation of the different transposon insertions in the F1 progeny is expected given the random integration of transposons into the genome, we observed that individuals in the cohort of three littermates shown have similar representations of the transposon pool, this is likely due to multiple copies of each individual transposon being present in the genome at different loci. We are able to verify that the nested transposon is intact, and where *SB11* transposase was used to mobilise the transposon, we can also verify the excision of the *SB* transposon from the flanking *PB* terminal repeats by PCR (Figure 1f).

### Validating cancer mutations by tumourigenesis *in vivo*

For *in vivo* tumourigenesis, we aged the mice with daily health monitoring for malignancy. As detailed in Table 1, chimaeric mice with both transposons and transposase expression presented histopathology-verified tumours at a higher rate than control mice that had transposons but no constitutive transposase expression (details of individual animals in Additional file 1: Table S2). In F1 progeny animals, the presence of a transposon expressing the dominant negative allele of p53 reduced the median tumour latency by 33.5 weeks; interestingly, these had lower incidence of tumours. This might be due to a lower general fitness in animals with this transposon, resulting in more animals being culled for non-malignancies. Given that transposon mobilisation is a cell independent somatic event and individual transposons are also represented in different pool combination and ES cell clones, we scored the occurrence of each candidate cDNA expression in different tumour types as a way to assess the potential of a given cDNA in contributing to tumourigenesis. We detected cDNA expression from the transposons in approximately half of the tumours assayed. In tumours without detectable cDNA expression, we are not able to distinguish between tumours that arose because of a transient effect of an oncogene (for example, the expression of a mutant kinase cDNA from a transposon might have contributed to the establishment of a clone but is then lost subsequently), versus tumours that arose due to other mutations occurring spontaneously. While the potential for remobilisation is an inevitable effect of the constitutive transposase activity, we reasoned that we should avoid any *a priori* assumptions on the suitable developmental stage or timing for mobilisation of the transposons. Also, the ability to incorporate *in vivo* selection pressure to retain transposon expression would provide a more stringent assay for validating the candidate cDNAs, while decreasing the potential passenger transposon insertions that might occur after a transient transposase mobilisation. Table 2 shows the occurrence for expression of each transposon in each tumour type.

**Table 1:** **Summary of** ***in vivo*** **tumourigenesis assay**

**Generation**	**Transposase**	**Transposons**	**Animals (n)**	**Animals with tumours (n)**	**Median tumour latency (weeks)**	**Animals with tumours (%)**
F0 chimaeras	+	+	21	8	85.5	38
F0 chimaeras	-	+	20	3	89.9	15
F1 animals	+	+	65	44	108.4	67
F1 animals	+	+ (p53.R270H)	20	6	74.9	30

**Table 2:** **Occurrence of transposon cDNA expression in different tumour types**

**cDNA**	**Lymphoma**	**Carcinoma**	**Blastoma**	**Sarcoma**	**Total**
CDK2.P45L	11	5	0	0	16
ERN1.S768F	8	4	0	0	12
LYN	5	6	0	0	22
ERN1	6	4	0	0	10
ITK	6	3	0	0	9
MAPK8.G171S	5	4	0	0	9
NTRK3	3	6	0	0	9
HCK.D378G	4	4	0	0	8
MAPK8	4	4	0	0	8
CDK2	4	3	0	0	7
MAPK8.G177R	3	4	0	0	7
MGC42105.P411T	5	2	0	0	7
DGKB	4	1	1	0	6
HCK	2	4	0	0	6
NTRK3.H677Y	4	2	0	0	6
YSK4	4	2	0	0	6
YSK4.E512V	3	2	0	0	5
MAPK9	3	1	0	0	4
MGC42105	3	1	0	0	4
ITK.P23L	2	1	0	0	0
NTRK3.R678Q	1	2	0	1	1
DGKB.D592Y	0	0	0	1	1
DGKB.G501S	0	0	0	1	1
DGKB.P432S	0	0	0	1	1
MAPK9.K56N	0	1	0	0	1
DGKB.K704E	0	0	0	0	0
DGKB.M111I	0	0	0	0	0
LYN.D385Y	0	0	0	0	0
MAPK9.V13M	0	0	0	0	0

**Figure 2:**
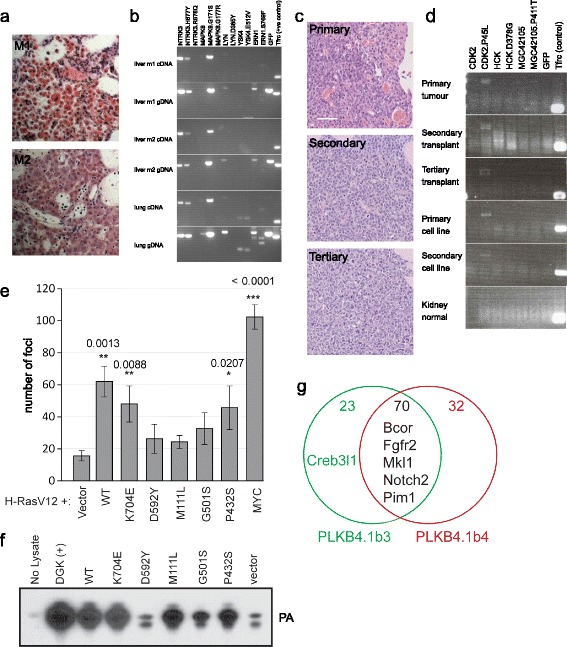
**Tumours generated in animals with transposons. (a)** An F_0_ chimaera (animal id PLKD4.1a) that presented with hepatocellular carcinoma in two liver lobes (m1 and m2), histological sections from both tumours visualised with haemotoxylin and eosin (H&E) stains. **(b)** Transposon expression (RT-PCR using cDNA) and representation (PCR using genomic DNA (gDNA)) in two tumour samples and normal lung. **(c)** An F_1_ animal (id PLKH1.4b) that presented with a solid lymphoma tumour (top panel), H&E histological section. Serial subcutaneous transplants of the tumour in NSG mice gave rise to secondary (middle panel) and tertiary tumours (lower panel). **(d)** Transposon expression of PLKH1.4b in the serially transplanted tumour, cell lines derived from the primary and secondary transplanted tumours, and normal kidney tissues. **(e)** Colony forming assay of different DGKB alleles in cooperation with HRAS.V12 using Ink/Arf mutant MEFs. MYC is a positive control. Error bars denote standard deviation, *P* value from two-tailed T-test compared to HRAS.V12 alone. **(f)** Relative activity of different DGKB alleles as measured by phosphatidic acid (PA) production. **(g)** Number of mutated genes unique to and shared between two regions of a single lymphoma that was exome-sequenced. Known cancer genes with mutations are shown.

Each occurrence of a cDNA’s expression in a tumour represents a potential context in which its expression contributed to tumourigenesis. In tumours, we often see loss of transposons and transposon cDNA expression compared to normal untransformed tissues. This bias can arise from clonal competition in dividing tumour cells, as transformed cells are likely to be represented by a few successful subclones. In contrast, the mixture of clones within normal tissues with different transposon insertion and expression profiles results in a net representation of most transposons from the germline. As an example, in an F_0_*PB* transposase expressing chimaera (animal id PLKD4.1a) with hepatocellular carcinoma (Figure 2a and b), the expression pattern in normal tissues suggests that stochastic and ectopic expression of many kinase alleles can be tolerated in normal tissues. In the tumour, only a subset of kinase transposon and cDNA expression is positively selected for and retained (Figure 2b).

To examine whether tumours retain transposon payload expression and have serial engraftment capacity, we transplanted fragments of 23 primary tumours by subcutaneous injection into immune-compromised NOD.Cg*-Prkdc*^*scid*^*Il2rg*^*tm1Wjl*^*/*SzJ (NSG) mice [18]. We observed that five (22%) of the tumours successfully engrafted in NSG mice. Of the five engrafted tumours, we attempted serial transplantation and four engrafted successfully again in NSG mice. In addition, efforts to create tumour cell lines from 34 primary tumours led to 13 tumour cell lines (38% success rate). As an example, from an F_1_ animal (id PLKH1.4b) with lymphoma, the tumour engrafted in NSG mice serially and maintained consistent histopathological features (Figure 2c). This animal had no transposon cDNA expression in sampled normal tissues and only CDK2.P45L expression in the lymphoma, the expression of the transposon cDNA was maintained through the serial transplants and also in cell lines derived from the tumour and the secondary engraftment (Figure 2d). Of the four tumours that underwent two serial engraftments, three showed retention of the transposon cDNA expression observed in the primary tumours.

The transposon construct with expression detected most frequently in tumours was the mutant allele of *Cyclin-dependent kinase 2* (CDK2.P45L, Table 1). The CDK2.P45L-expressing tumours were from animals generated by six independent ES cell clones with different pools of transposon insertions, pointing to the recurrence of CDK2.P45L expression as a result of positive selection. The CDK2.P45L mutation is a point substitution we observed once in a single glioblastoma patient where the conserved proline residue in the PSTAIRE interaction motif is replaced with leucine [14]. The PSTAIRE motif is the central helical motif in the interaction of CDK2 with its regulatory partner CYCLIN E1. Biochemical assays suggest that CDK2.P45L is unlikely to drive oncogenesis through an activating gain of function or deregulation mechanism, as the mutation disrupts the interaction between CDK2 and its regulatory binding partner CYCLIN E1 even though its kinase activity is retained [19]. Nevertheless, the recurrence and selective retention of expression in tumours (Figure 2a-d) observed here suggests that it can contribute a positive selective advantage to tumours *in vivo*.

In a separate pool, six transposons with alleles of *DGKB* (5 mutants, 1 wild-type) were introduced into the genomes of the ES cells, these mutations were observed in lung and melanoma cancer genomes (Figure 1c). The wild-type, K704E and P432S alleles have varying activity in transforming Ink/Arf-null mouse embryonic fibroblast (MEF) cells when expressed together with HRAS.V12 (Figure 2e). This transformation activity does not correlate with kinase activity of the protein, as the M111L allele retains kinase activity but does not cooperate with HRAS.V12 in transformation (Figure 2f). Remarkably, only the expression of the wild-type *DGKB* cDNA was recurrently recovered in multiple tumours (Table 1). While both substrate and product of *DGKB* (diacylglycerol and phosphatidic acid, respectively) play diverse roles in intracellular signalling [20], our data suggest that overexpressed *DGKB* can have oncogenic activity that is unrelated to its kinase activity, possibly through a scaffold or complex recruitment function given that the substitution mutants do not appear to contribute to tumourigenesis *in vivo* in this experimental context.

Bi-functional transposons designed with strong promoter/enhancer elements to drive ectopic gene expression and splice-acceptor-polyA signals to disrupt expression have been successfully used as insertional mutagens to identify cancer genes [7-10]. Unlike the insertional mutagenesis transposons used in those screens, the design of our cDNA delivery transposon does not contain any promoter/enhancer elements. While there is a possibility that splice acceptor’s promoter/enhancer-trapping activity can disrupt the expression of an endogenous tumour suppressor gene locus in a particular tumour sample, possibly resulting in hypomorphic gene function, the complete disruption of any particular gene function in a cell requires either a disrupting insertion followed by spontaneous loss of heterozygosity at the other copy, or two independent disrupting insertions at both copies of the gene in the chromosome pair. The basis of validating a candidate cDNA payload is based on observing recurrent expression across tumours from different animals, different founder animals derived from multiple ES cell clones, and with each tumour having a unique somatic profile of transposon insertions. Nevertheless, to assess whether the transposon-mediated delivery of the cDNAs contributes to a potential mutational load and to understand the cooperating mutations that could act together with the expressed exogenous kinases to drive tumourigenesis, we performed paired-end exome sequencing on tumour and normal matched samples from two animals that presented with lymphoma (animal ids PLKB4.1b and PLKE5.1a). Variants were called using customised CaVEMan and Pindel with output filtering (see Materials and methods). To validate the variants, we used Sanger sequencing for indels and pyrosequencing for substitutions. We validated 125 somatic mutations in PLKB4.1b and 28 somatic mutations in PLKE5.1a (Additional file 1: Tables S3, S4). Both animals were F_0_ chimaeras generated from transposase expressing ES cells, *PB* transposase in PLKB4.1b and *SB* transposase in PLKE5.1a. As *PB* transposition has been described to be largely precise and error-free in multiple species with no defined molecular signature [6,12,21,22], we are unable to quantify the relative contribution of transposon activity in the generation of the additional mutations in PLKB4.1b. However, given that *SB* excision results in a characteristic insertion of a short motif comprising the end of the transposon terminal repeat and a duplication of the TA dinucleotide insertion site [23,24], it is possible to estimate if transposition represents a significant mutation load. From the PLKE5.1a exome, none of the validated mutations exhibit the molecular footprint arising from *SB* transposition, suggesting that other additional drivers of mutations in tumours do occur and play the major role in the generation of these additional mutations. As the PLKB4.1b tumour was macroscopically heterogeneous, we sampled two different regions of the tumour for exome sequencing (sample ids PLKB4.1b3 and PLKB4.1b4). The two regions had 70 common mutations (75% and 68% of each region’s mutations respectively) that included five of the six known cancer genes mutated in both regions (Figure 2g, [25]). Both regions express CDK2.P45L cDNA. The regional exome sequencing shows that while a core set of mutations is likely to have contributed to the tumour, intra-tumour heterogeneity and genotypic divergence occurred in the life history of the tumour.

The *CDK2* and *DGKB* mutations highlight as examples that *in vivo* validation can provide novel information on the oncogenic activity of mutant alleles that would not be gleaned from biochemical or cell biology assays alone. In the case of CDK2.P45L, even though the mutant is unable to form a stable association with its cognate activating cyclin [19], the *in vivo* data showing positive selection for the expression of the mutant cDNA suggest that there could potentially be additional assembly factors that facilitate transient association between CDK2 and CYCLIN E1. For the DGKB mutant alleles, the uncoupling of kinase activity, transformation activity and *in vivo* representation in tumours underscores the critical importance for the multiplicity and depth of validation assays in functional cancer genomics. Without complementary functional characterization with *in vitro* and cell-based assays for the other mutations listed in Figure 1c, we are intentionally cautious in refraining from asserting whether the other mutations are validated or non-functional passenger mutations in the cancer genome. Even so, we note that our data presented here can serve to direct future investigations. Expression of both the wild-type and S768F mutant allele of *endoplasmic reticulum to nucleus signalling 1* (*ERN1*, also *IRE1*) are the second and fourth most frequently recovered transposon cDNAs in our study (Table 2), corroborating recent description of *ERN1* as playing key roles in tumour angiogenesis, growth and invasion [26,27]. In contrast, for the *v-yes-1 Yamaguchi sarcoma viral related oncogene homologue* (*LYN*) kinase that is implicated in a wide variety of cancers [28-30], while expression of the wild-type kinase is the third most frequent occurrence, we never recovered any tumours expressing the D385Y mutant (Table 2). This would suggest that the D385Y mutation is potentially a loss-of-function mutation, though this has to be characterised molecularly with biochemical and cell-based activity assays.

In this study, lymphomas and carcinomas were the predominant types of tumours generated. While the CDK2.P45L mutation was originally discovered in glioblastoma multiforme, its corresponding cDNA expression was detected in 11 lymphomas and five carcinomas in the *in vivo* tumourigenesis assay. This probably reflects a susceptibility of tissues to transformation and the underlying propensities of the promoter-transposase and transposon species combinations used here. A similar predisposition towards haematopoietic malignancies was observed in the first whole-body insertional mutagenesis screens with the *SB* transposon [7,8], and the predisposition can be varied by using a different transposon such as *PB* or by expressing the transposase from different promoters [31]. Indeed, from our relatively small cohort of F_0_ chimaeras derived from ES cells expressing transposase, a single *SB100* transposase-expressing animal presented with lymphoma. In comparison, six animals derived from ES cell clones expressing the *PB* transposase presented with lymphomas, hepatocellular carcinomas and a Wilms-like blastoma. Future iterations of this validation system could incorporate tissue-specific promoters or conditional ‘Lox-stop-Lox’ cassettes in the transposon to facilitate granular control for validating candidate cancer mutations in a tissue, developmental and temporal-specific manner.

With a larger animal cohort and tumour numbers, it also would be informative to explore in further studies whether co-expression of multiple transposon cDNA payloads could be indicative of synergistic genetic interactions in the pool. In this study, we have not taken into account the presence of any other transposon cDNA expression when looking at the recurrence of individual candidate cDNA payload, including the expression of the dominant-negative *Trp53* in some of our pools. We reasoned that it should be the selective retention of cDNA expression that is an indication of a candidate’s contribution to clonal selection and oncogenic potential. In addition to the selection forces we observed acting on the exogenous kinase cDNAs, the exome sequencing of the tumours show that these bona fide tumours can have additional mutations that are either prevalent or divergent within the tumour, exhibiting intratumour genotypic diversity and heterogeneity similar to that observed in the clonal evolutionary history of human tumours [32,33].

## Conclusions

We have shown here an efficient *in vivo* system for validating cancer genes that uses standard molecular biology and transgenic techniques. Several systems for elegant forward and reverse genetic screens have been engineered in mice that look at genes in specific tumour tissue types (that is, liver or brain) or in specific cancer processes such as metastasis [34-38]. While those systems allow for specific studies in particular tumour types and biological processes, the system described here allows for generalised whole-body *in vivo* validation of gain of function or neomorphic alleles that are observed in human cancer genomes. Given the crucial importance of balancing the requirements of *in vivo* validation with the resource-intensive nature of animal experiments, the system described here is advantageous as a generalised *in vivo* validation step in functional cancer genomics. The identification or validation of genes *in vivo* should be complemented by molecular and cellular characterisation. With our collection of kinase mutations that have not been previously characterised *in vivo*, we show here for the P45L mutation in *CDK2* and the *DGKB* alleles that *in vivo* contexts can reveal phenotypic divergences and nuances when compared to standard cell-based or biochemical assays. Compared to our previous experimental system that required the construction of large bacterial artificial chromosomes for the transposon gene arrays [13], the procedures for generating these animals are relatively simple, providing an easy means of polygenic validation in multiple somatic tissue types per animal line that is not previously accessible. This transposon-based *in vivo* system allows us to isolate somatic tumours where the oncogene candidates are expressed in a susceptible tissue microenvironment and at levels that are clonally advantageous, complementing existing *in vitro* and cell-based assays for validating and characterising genes. By using a system where the candidate mutation has to be selectively retained, this system also provides a means of generating cell lines useful for further characterising functional requirements and roles of the mutations. This is particularly advantageous as cancer genes and mutation candidates from large scale sequencing studies are often not preserved within a functional context in patient-derived cell lines. While the overall throughput of validation described here averages a little less than a dozen candidate cDNAs per animal line, the relative simplicity of the techniques described here is scalable with the number of candidates for validation by increasing the number of animal lines. The *in vivo* validation system described here should prove to be a useful addition to the current suite of functional cancer genomics tools.

## Materials and methods

### Transposon constructs

The piggyBac transposase construct and components of the nested PB-SB transposon vector were cloned as previously described [12,13]. Kinase mutations were generated using the QuikChange Lightning Site-Directed Mutagenesis Kit (Stratagene) and cDNA clones from the IMAGE consortium. NTRK3 cDNA was a gift from B.D. Nelkin [39]. The cDNAs and the 60-basepair unique sequence tag were added by PCR cloning (KOD, Takara) into the transposon vector (See Additional file 1: Table S1 for tag sequences).

### Cell culture

Both AB2.2 and JM8A3 mouse ES cells were grown in standard M15 media on SNL76/7 feeder cells [40]. M15 comprises Knockout DMEM (GIBCO), 15% Fetal Bovine Serum (Invitrogen), 1× Pencillin, Streptomycin and Glutamine (GIBCO), 1× Non-Essential Amino Acids (GIBCO), 0.1 mM β-mercaptoethanol (Sigma), 1,000 U/mL human LIF (Millipore). Tumour derived cell lines were cultured in RPMI with 10% Fetal Bovine Serum (Invitrogen), 1× Pencillin, Streptomycin and Glutamine (GIBCO), 1× Non-Essential Amino Acids (GIBCO).

### Manipulation of mouse ES cells

To electroporate mouse ES cells, cells were harvested by trypsinisation, washed in PBS and resuspended to 10^7^ cells/mL. For each mL of cells, 30 μg total of pooled transposon constructs of equal quantity were co-electroporated with 10 μg of CAGG-PBase transposase helper plasmid and 30 ng of *PB-SB-pGK-Neo* co-selection marker on a Biorad GenePulser. The electroporation settings are 230 V, 500 μF with expected time constant between 5.6 and 8.0. In all transposon pools, *PB-SB-ires-eGFP* is a negative control in the pool. Selection for clones containing transposon using *PB-SB-pGK-Neo* co-selection was done in M15 media supplemented with 125 μg/mL Geneticin (GIBCO).

### Animal work

All animal-related protocols and care was provided in accordance with the Animal (Scientific Procedures) Act 1986. To generate animals, chimaera animals were generated by injecting ES cells into blastocysts as per standard protocols. For the tumourigenesis assay, animals are aged and monitored daily for signs of malignancy. Animals exhibiting signs of poor health or distress are euthanized by exposure to rising CO_2_ concentration, followed by necropsy and histolopathological analyses of tissues.

For serial tumour transplant, tumour samples were harvested immediately after the animal was culled. Tumour tissue is briefly surface sterilised in 70% ethanol and immediately washed in PBS, followed by mincing with scalpels. Fine tumour mince is injected subcutaneously into the flanks of NSG mice with a 21-gauge needle and syringe.

### Histology

Tissues were fixed overnight in 10% formalin (Sigma), dehydrated and cleared for paraffin embedment as per standard histology protocols. The paraffin blocks were cut at 5 μm thickness and stained with haematoxylin and eosin as per standard protocols. All culled animals were scored for presence of tumour and tumour type(s) as applicable, independently of the detection of transposon and cDNA expression without randomisation or blinding.

### Detecting transposon and payload cDNA expression

Both genomic DNA and RNA are isolated from cells or tissues using an AllPrep kit (Qiagen) where fresh tissues were available, this was not done in cases such as found dead animals where the tissues were degraded. For reverse transcriptase reaction, cDNA was synthesised from extracted RNA using Superscript III (Invitrogen). PCR was done on standard conditions using Extensor (Thermo), primers pairs are listed in Additional file 1: Table S5. The primer pair for *Transferrin receptor* (*Tfrc*) positive control spans an intron, allowing detection of any genomic DNA contamination in cDNA PCR and vice versa.

### Cell line derivation

Fresh tumour mince is divided between three 35 mm tissue culture dishes and incubated for 16 h in RPMI media supplemented with 30, 100 and 300 U/mL collagenase II (GIBCO). Cells from all three dishes are collected the next day, centrifuged at 200 rcf for 5 min and washed in RPMI media to remove the collagenase. The resulting cell suspension is seeded into 75 cm^2^ flasks, media changed every 2 to 4 days until the cells are approximately 80% confluent and ready for subculturing.

### Kinase assay

DGKB kinase activity was assessed by measuring the abundance of phosphatidic acid (PA) as a reflection of cellular DGK activity in protein lysates, as described previously [41]. Briefly, lysates from 293 cells transfected with the wild-type and indicated DGKB mutants were separated by thin layer chromatography, PA abundance was analysed by autoradiography. Empty vector and Bacterial DGK were used as negative and positive controls, respectively.

### Transformation assay

*Ink4a/Arf-*deficient primary murine embryonic fibroblasts (MEFs) were plated in DMEM containing 10% FBS at a density of 8 × 10^5^ cells per 10 cm, 16 h before transfection. For RAS cooperation, 1.5 μg HRAS(Val 12) vector was co-transfected with 6.5 μg pEF-Dest51-LacZ control vector, MYC or the indicated DGKB variants in pEF-Dest51 using Lipofectamine2000 (Invitrogen) following the manufacturer’s instructions. The total amount of transfected DNA was kept constant at 7.5 μg, and transfections were done in duplicate three times. At 48 h after transfection, each transfected 10-cm plate was equally split into three 10-cm plates and incubated for 10 days, during which media was refreshed twice. Cells were washed, fixed in 10% formalin and stained with Giemsa solution (Sigma) for 10 min at room temperature for foci quantification. Two-tailed *t-*test calculations were performed using Prism 4 (Graphpad).

### Exome sequencing

Sequencing was based on exome capture and performed using 76 basepair paired-end reads on the Illumina GAIIx platform. The sequencing data are deposited into the European Nucleotide Archive under study accession number ERP000896. Mapping of the sequence data was done using BWA [42]. Variant calling was done using CaVEMan (Cancer Variants through Expectation Maximisation) [43] for substitutions and Pindel [44] for insertions and deletions, modifications to the variant callers and subsequent filtering were as previously described [45]. These algorithms identified somatic variants in the tumour samples compared to matched normal sample for the same animal.

## Additional file

Additional file 1: Table S1. Tag sequences. **Table S2.** Details of tumour bearing mice. **Table S3.** Validated mutations from animal PLKB4.1b. **Table S4.** Validated mutations from animal PLKE5.1a. **Table S5.** Genotyping primers.
