# Supplementary material for: Polygenic in vivo validation of cancer mutations using transposons
Source: Genome Biol. 2014 Sep 27;15(9):455. doi: 10.1186/s13059-014-0455-6 (PMC4210617; doi:10.1186/s13059-014-0455-6)
Supplement: Additional file 1: Table S1. — Tag sequences. Table S2. Details of tumour bearing mice. Table S3. Validated mutations from animal PLKB4.1b. Table S4. Validated mutations from animal PLKE5.1a. Table S5. Genotyping primers. [file 13059_2014_455_MOESM1_ESM.pdf]

Table S1. Tag sequences

cDNA	Unique sequence tag
CDK2	CGAGTGACTAGATACGCTATTCACACTGCGGAATTAGCGCCGCTAGTATTCGATATGGGT
CDK2.P45L	CTATGACGGAGCAGATACCGCCCGTTAGTTAAGATTCTCGCAACTCGTCGTAAGGTAGCG
DGKB	AGTACACCCGACCAGTGAAGCCTAACTAGAGATATGCGTCACTTGTTAAGCAGGACTGAG
DGKB.D592Y	TCAGCTTCGATAGCCGAGGTGCCACGCTACTAAGTTTATCGGCTAGATTAACGTACTGTC
DGKB.G501S	CCTCTAGGTGTACGGAGCTTTTTAGTCCGCGACGGGATCTGCGCATCGCAAATCCGAGAA
DGKB.K704E	GACGCGCTCGTAACTCATAGTTCATTGGACGCAGTCACGGCGAGTTAATCTCAGCGAACG
DGKB.M111I	GAGATCACTCGAACTTATCCGCGTGATAAGCATTCCGCAGTATAGAGACGCACCCGAAGC
DGKB.P432S	GGGACTCTAGTAGTATGCTTTTCGGTACGCGATAGCAGCTACGCTATGAGGATTGATCGT
ERN1	AATGTAGACTCACGCACGCGGCCTGCCATCAATTCGATTAATACTCGTTAGCAGGTTTGG
ERN1.S769F	ATGAGACGGCTAGTACGCCTGACAGCGTTACAAGTACCTACGGATCTAACTACACTGTGC
HCK	TATGACCTCGCACTAGCGTGGACTACCTAATACGACGTGCTAGTTCGGTACATTGCGCTG
HCK.D399G	AGGTCAGACAGCATTCGCTCACTACCGGCTACGACTTGGTACCCGAGCGTTGATAATTCT
ITK	TCCAGTGGCTAAGTGTTCCGTATCCTCGGAGACGCGATTGTACGATCCGGCATGTCGATG
ITK.P23L	CACGTTTGCGAATAGGTATCGAGATCCACGCAATCGCATACGACGATGCTTATAGACTCT
LYN	ATATCGACCATCCGCGTAAGATATTATCGGTGACGAGCGTACACGATGACAATGCCTGGA
LYN.D385Y	GCAGCTACTCGCACTGATTGAATCGGTACTAAGTCGGTTCACGTCGTACTCTAGCTCGTT
MAPK8	GAGTGCATTACAGATTACCGAATCAGTATGAAGCGAGCGCACACCGAGCCGATATTCGTG
MAPK8.G171S	GTACGTGTCGGCACATCGTTATCGCAACTAACGCGCTATACAGTAGACCCGACTCGTTTG
MAPK8.G177R	CCTTGGACGGTATATGATGTACGCTTACGCGACTTTCGGTTATCGGACCAGCAGGCTAAG
MAPK9	ATTAGCAGCGCCATTACGCGGTACACAGATACGCGCTCAGCAGCGACTGCAACTATTGTA
MAPK9.K56N	GGTTATATCACATCCTTCGCACTATCTATCGAGCGGGAGTATAGAAGTATAAGTGCGCCG
MAPK9.V13M	GGTACGAGATAAGTAGATCCGCTACATCGTAATTGTCCTCGCCGCAGTGACAATAATTCA
MGC42105	GCCTCACTATTCACGTTATGCACTCGTAACTATACCGAAGACTGTACTACTCGCTCGATT
MGC42105.P411T	TTAGCACGTAGAGCATTGCGACATCGGGTAAACGCCTCGAGGCTTAATCAAACCCGCTAA
NTRK3	GGCTTAACTATCACGATCAGGGCATACCGACAACGATTCAGCCCGCTAACGAAAGGGTAA
NTRK3.H677Y	CTGTGCGCGAAAGCAACGAAAATGATAGGTAATCGGACCGCACGAATCGGAATTGACGTA
NTRK3.R678Q	TAATCCGCACGAGCTGCTAGCTCATGTACGTCGAGGAGCTATTTAGCGTGCAGCCGACCT
p53.R270H	GCGGCCTTAGGATTGATATTATCATGGTGGACATCGTTTGCCGTATAGCTTAATAGCCAG
YSK4	GCCCTAGCCCGGATTAGTTTCCGGTAACTTCGTATAAGAGCTAATGCGGTTTAAGCGATG
YSK4.E21V	CCTCTAAGCTCACGTTATAGGTACCCATCGGCACGTTTAGTTAACGCTGATGCACCGGCT

Table S2. Details of tumour bearing mice

Mouse ID	Generation	Genotype	Age at death (weeks)	Tumour type	Kinase alleles expressed in tumours	ES cell ID[1]	Transposase in ES cells[1]
PLKB4.1b	F0 chimaera	Transposase + Transposons	112.8	Lymphoma	CDK2.P45L. CDK2.P45L expression retained in primary cell line	TM201	PB
PLKD1.1a	F0 chimaera	Transposase + Transposons	109.5	Hepatocellular carcinoma	LYN, ERN1	TM402	PB
PLKD3.1a	F0 chimaera	Transposase + Transposons	67.4	Hepatocellular carcinoma	NTRK3	TM402	PB
PLKD4.1a	F0 chimaera	Transposase + Transposons	68.2	Hepatocellular carcinoma	NTRK3, NTRK3.H677Y, MAPK8.G171S, LYN	TM402	PB
PLKE5.1a	F0 chimaera	Transposase + Transposons	99.5	Lymphoma, follicular	YSK4, YSK4.E512V. Kinase cDNA expression lost in primary cell line	TM401	SB100
PLKD3.1c	F0 chimaera	Transposase + Transposons	86.5	Lymphoma	None detected	TM402	PB
PLKB4.1a	F0 chimaera	Transposase + Transposons	84.5	Wilms tumour like	None detected	TM402	PB
PLKD7.1a	F0 chimaera	Transposase + Transposons	71.8	Atypical Lipoma	None detected	TM402	PB
PLKK6.1a	F0 chimaera	Transposons only	96.1	Lymphoma	Not done	T24AC6	
PLWG3.1c	F0 chimaera	Transposons only	89.9	Lymphoma	Not done	T25ABE1	
PLWG6.1b	F0 chimaera	Transposons only	85.5	Hepatocellular carcinoma	Not done	T24AC6	
PLKH1.3c	F1	Transposase + Transposons	67.4	B-cell Lymphoma	CDK2.P45L	TM201	PB
PLKL7.2c	F1	Transposase + Transposons	67.6	Lymphoma	MGC42105, MGC42105.P411T, ITK, NTRK3, NTRK3.H677Y, MAPK8.G171S, LYN, YSK4, ERN1, ERN1.S768F	T24AC6	
PLKH1.4b	F1	Transposase + Transposons	104.6	Lymphoma	CDK2.P45L. CDK2.P45L expression retained in cell line from primary tumour and secondary tumour	TM201	PB
PLKJ2.1c	F1	Transposase + Transposons	105.8	Hepatocellular carcinoma	NTRK3	TM401	SB100
PLKH1.2c	F1	Transposase + Transposons	117.4	Burkitt-like lymphoma	CDK2.P45L	TM201	PB
PLKH1.2d	F1	Transposase + Transposons	117.6	Lymphoma	CDK2.P45L. CDK2.P45L retained in secondary transplanted tumour	TM201	PB
PLKJ1.1a	F1	Transposase + Transposons	112.9	Lymphoma	ERN1, ERN1.S768F	TM401	SB100
PLKL5.2b	F1	Transposase + Transposons	87.1	Lymphoma	CDK2.P45L, HCK, HCKD378G, MGC42105, MGC42105.P411T, MAPK9, ITK, ITK.P23L, NTRK3.H677Y, MAPK8, MAPK8.G171S, MAPK8.G177R, LYN, ERN1, ERN1.S768F	T24AF11	
PLKH1.4c	F1	Transposase + Transposons	125.1	Lymphoma	CDK2.P45L	TM201	PB
PLKL5.2a	F1	Transposase + Transposons	91.2	Lymphoma	CDK2, CDK2.P45L, HCK, HCK.D378G, MGC42105.P411T, MAPK9, ITK, ITK.P23L, NTRK3.H677Y, NTRK3.R678Q, MAPK8, MAPK8.G171S, MAPK8.G177R, LYN, ERN1.S768F	T24AF11	
PLKL7.4d	F1	Transposase + Transposons	90.5	Lung adenocarcinoma	CDK2, CDK2.P45L, HCK.D378G, MGC42105, ITK, NTRK3, NTRK3.H677Y, MAPK8, MAPK8.G171S, YSK4.E512V	T24AC6	
PLKL7.4c	F1	Transposase + Transposons	93.9	Lymphoma	CDK2, CDK2.P45L, MGC42105.P411T, ITK, NTRK3, MAPK8, MAPK8.G171S, LYN, YSK4.E512V, ERN1.S768F	T24AC6	
PLKL7.4a	F1	Transposase + Transposons	94.1	Lymphoma	CDK2.P45L, MGC42105.P411T, MAPK9, ITK, NTRK3, NTRK3.H677Y, MAPK8, YSK4.E512V, ERN1, ERN1.S768F	T24AC6	
PLKJ1.1c	F1	Transposase + Transposons	122.9	Hepatocellular carcinoma	MAPK8.G177R	TM401	SB100
PLKL5.1a	F1	Transposase + Transposons	109.4	Lymphoma	CDK2, CDK2.P45L, HCK.D378G, MGC42105, MAPK8.G171S, MAPK8.G177R, YSK4, ERN1, ERN1.S768F. Derived cell line retains expression of CDK2, CDK2.P45L, HCK.D378G, MGC42105, MAPK8.G171S, MAPK8.G177R, YSK4, ERN1, ERN1.S768F	T24AF11	
PLKJ2.2h	F1	Transposase + Transposons	69.4	Lymphoma	None detected	TM401	SB100
PLKG2.1f	F1	Transposase + Transposons	127.4	Primitive neuroectodermal tumor	None detected	TM301	PB
PLKJ2.2a	F1	Transposase + Transposons	106.1	Lung adenocarcinoma	None detected	TM401	SB100
PLUD1.1h	F1	Transposase + Transposons	82.8	Lymphoma	None detected	TM401	SB100
PLKJ2.1b	F1	Transposase + Transposons	80.2	Mucoepidermoid carcinoma	None detected	TM401	SB100
PLKH1.2b	F1	Transposase + Transposons	117.6	Angiosarcoma	None detected	TM201	PB
PLKG2.1a	F1	Transposase + Transposons	115.9	Lymphoma	None detected	TM301	PB
PLKJ1.3d	F1	Transposase + Transposons	109.2	Liver angiosarcoma.	None detected	TM401	SB100
PLKJ1.4a	F1	Transposase + Transposons	107.5	Hepatocellular carcinoma	None detected	TM401	SB100
PLKH1.2a	F1	Transposase + Transposons	130.4	Hepatocellular carcinoma	None detected	TM201	PB
PLKJ2.2d	F1	Transposase + Transposons	100.4	None detected	None detected	TM401	SB100
PLUD1.1c	F1	Transposase + Transposons	121.2	Lung papillary carcinoma	None detected	TM401	SB100
PLKJ2.2f	F1	Transposase + Transposons	114.9	Adenocarcinoma	None detected	TM401	SB100
PLKJ1.3g	F1	Transposase + Transposons	115.4	Carcinoma, lymphoma.	None detected	TM401	SB100
PLKH1.5a	F1	Transposase + Transposons	96.8	Lymphoma	None detected	TM201	PB
PLUD1.1f	F1	Transposase + Transposons	96.5	Ovarian serous papillary carcinoma	Not done	TM401	SB100
PLKG2.1g	F1	Transposase + Transposons	122.1	Lymphoplasmacytic lymphoma	Not done	TM301	PB
PLKL5.2c	F1	Transposase + Transposons	98.4	Lymphoma	Not done	T24AF11	
PLKL7.2a	F1	Transposase + Transposons	97.8	Lymphoma	Not done	T24AC6	
PLKJ1.1e	F1	Transposase + Transposons	115.1	Lymphoma	Not done	TM401	SB100
PLKL7.1d	F1	Transposase + Transposons	68.5	Lymphoma	Not done	T24AC6	
PLKG2.1e	F1	Transposase + Transposons	109.2	Lymphoma	Not done	TM301	PB
PLUD2.1a	F1	Transposase + Transposons	135.8	Lymphoma	Not done	TM401	SB100
PLKL7.2b	F1	Transposase + Transposons	69.6	Lymphoma	Not done	T24AC6	
PLKL7.1b	F1	Transposase + Transposons	72.8	Harderian gland adenoma	Not done	T24AC6	
PLKJ2.1h	F1	Transposase + Transposons	111.6	Lymphoma	Not done	TM401	SB100
PLKJ2.2c	F1	Transposase + Transposons	125.4	Lymphoma	Not done	TM401	SB100
PLKL7.4b	F1	Transposase + Transposons	91.5	Hepatocellular carcinoma	Not done	T24AC6	
PLKJ1.4e	F1	Transposase + Transposons	124.8	Lymphoma	Not done	TM401	SB100
PLKP25.1e	F1	Transposase + Transposons (including p53.R270H)	61.8	Hepatocellular carcinoma	CDK2, CDK2.P45L, HCK, ITK, NTRK3, NTRK3.R678Q, MAPK8, MAPK8.G171S, MAPK8.G177R, LYN, YSK4, YSK4.E512V, ERN1, ERN1.S768F, P53.R270H	T25BBD9	
PLKP13.1b	F1	Transposase + Transposons (including p53.R270H)	69.1	Adenoid cystic carcinoma	CDK2.P45L, HCK, HCK.D378G, MGC42105.P411T, NTRK3.R678Q, MAPK8, MAPK8.G171S, LYN, ERN1.S768F and p53.R270H. Primary cell line expresses CDK2.P45L, HCK, HCK.D378G, MGC42105.P411T, MAPK9.V13M, NTRK3, MAPK8, MAPK8.G177R, ERN1.S768F, p53.R270H.	T25ABE10	

PLKP3.4h	F1	Transposase + Transposons (including p53.R270H)	72.9	Lung adenocarcinoma	CDK2.P45L, MAPK9, LYN, ERN1.S768F	T25ABE1	
PLKP3.3a	F1	Transposase + Transposons (including p53.R270H)	79.2	Hepatocellular carcinoma	CDK2, CDK2.P45L, HCK, HCK.D378G, MGC42105.P411T, MAPK9.K56N, ITK, ITK.P23L, NTRK3, MAPK8, MAPK8.G177R, YSK4, ERN1, ERN1.S768F	T25ABE1	
PLKP7.2b	F1	Transposase + Transposons (including p53.R270H)	83.5	Lymphoma	CDK2, CDK2.P45L, HCK.D378G, ITK, LYN, YSK4, ERN1, ERN1.S768F	T25BBD9	
PLKP24.2b	F1	Transposase + Transposons (including p53.R270H)	76.8	Hepatocellular carcinoma	HCK, HCK.D378G, LYN, ERN1	T25BBD9	
PLDG23.1d	F1	Transposase + Transposons	81.9	Lymphoma	DGKB	DG1C5	
PLDG19.1e	F1	Transposase + Transposons	114.8	Lymphoma	DGKB. DGKB expression retained in primary cell line	DG2D3	
PLDG19.1b	F1	Transposase + Transposons	115	Hepatocellular carcinoma	DGKB	DG2D3	
PLDG19.1d	F1	Transposase + Transposons	116.9	Diffuse large B-Cell lymphoma	DGKB	DG2D3	
PLDG37.1a	F1	Transposase + Transposons	47.6	Primitive neuroectodermal tumor	DGKB	DGJA8	
PLDG19.1f	F1	Transposase + Transposons	121.4	Lymphoma	DGKB	DG2D3	
PLDG8.1a	F0 chimaera	Transposase + Transposons	57.6	Sarcoma	DGKB.D592Y, DGKB.G501S, DGKB.P432S	DG1C5	
PLDG17.1e	F1	Transposase + Transposons	111.4	Leiomyosarcoma	None detected	DG2D3	
PLDG23.1f	F1	Transposase + Transposons	86.4	Lymphoma	Not done	DG1C5	
PLDG19.1a	F1	Transposase + Transposons	102.8	Lymphoma	Not done	DG2D3	
PLDG19.1g	F1	Transposase + Transposons	102.8	Lymphoma, glioblastoma	Not done	DG2D3	
PLDG17.1b	F1	Transposase + Transposons	105.5	Lung adenocarcinoma	Not done	DG2D3	

1. Kinase alleles (and transposase, if present) represented in the ES cells used to generate the original founder F0 animals are as follows

TM201	Rosa26-PBase	CDK2, CDK2p.P45L, HCK, HCKp.D378G, MGC42105, MGC42105p.P411T
TM301	Rosa26-PBase	MAPK9, MAPK9p.V13M, MAPK9p.K56N, ITK, ITKp.P23L
TM401	Rosa26-SB100	NTRK3, NTRK3.H677Y, NTRK3.R678Q, MAPK8, MAPK8.G171S, MAPK8.G177R, LYN, LYN.D385Y, YSK4, YSK4.E512V, ERN1, ERN1.S769F
TM402	Rosa26-PBase	NTRK3, NTRK3.H677Y, NTRK3.R678Q, MAPK8, MAPK8.G171S, MAPK8.G177R, LYN, LYN.D385Y, YSK4, YSK4.E512V, ERN1, ERN1.S769F
T24AC6		CDK2, CDK2.P45L, HCK, HCK.D378G, MGC42105, MGC42105.P411T, MAPK9, MAPK9.V13M, ITK, NTRK3, NTRK3.H677Y, MAPK8, MAPK8.G171S, MAPK8.G177R, LYN, YSK4.E512V, ERN1, ERN1.S768F
T24AF11		CDK2, CDK2.P45L, HCK, HCK.D378G, MGC42105, MGC42105.P411T, MAPK9.V13M, ITK, ITK.P23L, NTRK3, NTRK3.H677Y, NTRK3.R678Q, MAPK8, MAPK8.G171S, MAPK8.G177R, LYN, YSK4, ERN1.S768F
T25ABE1		CDK2, CDK2.P45L, HCK, HCK.D378G, MGC42105, MGC42105.P411T, MAPK9.V13M, MAPK9.K56N, ITK, ITK.P23L, NTRK3, NTRK3.R678Q, MAPK8, MAPK8.G171S, MAPK8.G177R, LYN, LYN.D385Y, YSK4, YSK4.E512V, ERN1, ERN1.S768F
T25ABE10		CDK2, CDK2.P45L, HCK, HCK.D378G, MGC42105.P411T, MAPK9.V13M, MAPK9.K56N, ITK, NTRK3, NTRK3.R678Q, MAPK8, MAPK8.G171S, LYN, YSK4, ERN1, ERN1.S768F
T25BBD9		CDK2, CDK2.P45L, HCK.D378G, MGC42105.P411T, MAPK9, MAPK9.K56N, ITK, ITK.P23L, NTRK3.H677Y, MAPK8, MAPK8.G171S, LYN, YSK4, ERN1, ERN1.S768F
DG1C5		DGKB, DGKB.D592Y, DGKB.M111I, DGKB.P432S, DGKB.G501S, DGKB.K704E
DG2D3		DGKB, DGKB.D592Y, DGKB.M111I, DGKB.P432S, DGKB.G501S, DGKB.K704E
DGJA8		DGKB, DGKB.D592Y, DGKB.G501S, DGKB.K704E

Table S3. Validated mutations from animal PLKB4.1b

Sample ID	Variant ID	Chromosome	Position	WT	Mut	Change	Ensembl gene ID	Gene name	Description	Mutation consequence
PLKB4.1b3	39392547	1	19094468	C	G		ENSMUSG00000042596	Tfap2d	p.S21R	Missense
PLKB4.1b3	39392640	1	58351208	G	A		ENSMUSG00000079554	Aox3l1	p.A247T	Missense
PLKB4.1b3	39392646	1	59042200	A	C		ENSMUSG00000026027	Stradb	p.D54A	Missense
PLKB4.1b3	39392101	1	82285966	C	t		ENSMUSG00000055980	Irs1	p.R368H	Missense
PLKB4.1b3	39392564	1	113756251	A	C		ENSMUSG00000038702	Dsel	p.Y1044D	Missense
PLKB4.1b3	39392883	1	174149628	T	C		ENSMUSG00000007122	Casq1	p.E59G	Missense
PLKB4.1b3	39399659	2	26881500	C	T		ENSMUSG00000036067	Slc2a6	p.G127D	Missense
PLKB4.1b3	39399882	2	34868531	G	A		ENSMUSG00000026874	Hc	p.A1076V	Missense
PLKB4.1b3	39399905	2	40716207	C	T		ENSMUSG00000049252	Lrp1b	p.R3100H	Missense
PLKB4.1b3	39399393	2	52578993	T	G		ENSMUSG00000055371	Stam2	p.K48N	Missense
PLKB4.1b3	39399941	2	86349512	T	G		ENSMUSG00000060742	Olfr1076	p.Y299D	Missense
PLKB4.1b3	39399943	2	86517195	C	G		ENSMUSG00000075175	Olfr1086	p.Q98H	Missense
PLKB4.1b3	39399431	2	88586532	A	G		ENSMUSG00000075117	Olfr1198	p.I171T	Missense
PLKB4.1b3	39399440	2	91823486	T	G		ENSMUSG00000027230	Creb3l1	p.K454N	Missense
PLKB4.1b3	39399358	2	110571699	C	T		ENSMUSG00000050808	Muc15	p.P108S	Missense
PLKB4.1b3	153047091	2	136372637			G	ENSMUSG00000074771	Ankrd5	p.G262fs*9	Frameshift deletion
PLKB4.1b3	39400004	2	174253467	A	G		ENSMUSG00000016256	Ctsz	p.M276T	Missense
PLKB4.1b3	39393742	3	38856044	G	C		ENSMUSG00000046743	Fat4	p.V2124L	Missense
PLKB4.1b3	39393985	3	72854701	T	C		ENSMUSG00000048304	Slitrk3	p.H220R	Missense
PLKB4.1b3	39394029	3	97951233	T	G		ENSMUSG00000027878	Notch2	p.S2403A	Missense
PLKB4.1b3	39394695	4	14902332	T	C		ENSMUSG00000040536	Necab1	p.D232G	Missense
PLKB4.1b3	39394754	4	104712702	T	G		ENSMUSG00000028518	Prkaa2	p.E306D	Missense
PLKB4.1b3	153045946	4	135999157			CA	ENSMUSG00000070687	Htr1d	p.T264fs*16	Frameshift deletion
PLKB4.1b3	39395392	5	4099235	T	C		ENSMUSG00000001467	Cyp51	p.E214G	Missense
PLKB4.1b3	39396440	6	41721935	T	C		ENSMUSG00000045479	Olfr459	p.D121G	Missense
PLKB4.1b3	39396797	6	54996424	A	C		ENSMUSG00000029777	Gars	p.K70T	Missense
PLKB4.1b3	39396799	6	55228153	A	G		ENSMUSG00000038022	Fam188b	p.D511G	Missense
PLKB4.1b3	39396812	6	56907279	A	C		ENSMUSG00000071893	Vmn1r4	p.D258A	Missense
PLKB4.1b3	153058596	6	125121921			C	ENSMUSG00000038252	Ncapd2	p.A976fs*4	Frameshift insertion
PLKB4.1b3	39396740	6	132907367	T	A		ENSMUSG00000057699	Tas2r131	p.K166*	Nonsense
PLKB4.1b3	39398882	7	17740146	G	T		ENSMUSG00000053228	Ceacam3	p.E169*	Nonsense
PLKB4.1b3	39398587	7	25917455	A	C		ENSMUSG00000008496	Pou2f2	p.M6R	Missense
PLKB4.1b3	39398761	7	50915345	C	T		ENSMUSG00000030474	Siglece	p.R53K	Missense
PLKB4.1b3	39398773	7	51784776	C	A		ENSMUSG00000008193	Spib	p.C100F	Missense
PLKB4.1b3	39398774	7	51784853	T	C		ENSMUSG00000008193	Spib	p.N84D	Missense
PLKB4.1b3	39398402	7	70244531	C	T		ENSMUSG00000030525	Chrna7	p.R363H	Missense
PLKB4.1b3	39399122	7	110076706	G	A		ENSMUSG00000073963	Olfr572	p.R188H	Missense
PLKB4.1b3	39399000	7	137321263	G	A		ENSMUSG00000030849	Fgfr2	p.Q616*	Nonsense
PLKB4.1b3	39389805	8	14822586	G	A		ENSMUSG00000047495	Dlgap2	p.R689H	Missense
PLKB4.1b3	39390082	8	28519809	G	A		ENSMUSG00000031491	Chrna6	p.R87C	Missense
PLKB4.1b3	39390016	8	53282407	A	G		ENSMUSG00000052825	Gm9892	p.S2P	Missense
PLKB4.1b3	39389967	8	88164565	T	C		ENSMUSG00000036902	Neto2	p.Y511C	Missense
PLKB4.1b3	153050793	8	111774830			A	ENSMUSG00000057387	4922502B01Rik	p.L201fs?	Frameshift deletion
PLKB4.1b3	39400168	9	36342022	G	A		ENSMUSG00000074448	Gm5615	p.H73Y	Missense
PLKB4.1b3	39400187	9	38635654	A	G		ENSMUSG00000044798	Olfr923	p.Y126C	Missense
PLKB4.1b3	153052237	9	39509846			G	ENSMUSG00000062121	Olfr149	p.D171fs*2	Frameshift deletion
PLKB4.1b3	39400141	9	53181593	C	T		ENSMUSG00000034584	Exph5	p.T623I	Missense
PLKB4.1b3	39400249	9	73780833	A	C		ENSMUSG00000062151	Unc13c	p.L181R	Missense
PLKB4.1b3	39400687	9	108016984	T	G		ENSMUSG00000032589	Bsn	p.Q1300P	Missense
PLKB4.1b3	39388747	10	23720702	G	T		ENSMUSG00000069705	Taar7b	p.A320S	Missense
PLKB4.1b3	39388755	10	26844287	C	A		ENSMUSG00000019899	Lama2	p.V1682F	Missense
PLKB4.1b3	153047852	11	53584794			C	ENSMUSG00000018899	Irf1	p.P2fs*7	Frameshift deletion
PLKB4.1b3	153047857	11	55086649			C	ENSMUSG00000055333	Fat2	p.G3094fs*8	Frameshift deletion
PLKB4.1b3	39396236	11	60310491	G	A		ENSMUSG00000042678	Myo15	p.R763H	Missense
PLKB4.1b3	153048037	11	77728486			G	ENSMUSG00000055045	Gm11190	p.P41fs*23	Frameshift deletion
PLKB4.1b3	39396051	11	87381186	G	A		ENSMUSG00000020486	Sept4	p.A295T	Missense
PLKB4.1b3	39396073	11	90499631	T	G		ENSMUSG00000020544	Cox11	p.L43R	Missense
PLKB4.1b3	39396325	11	98338239	A	T		ENSMUSG00000018168	Ikzf3	c.823+2T>A	Essential splice
PLKB4.1b3	39396326	11	98338271	G	A		ENSMUSG00000018168	Ikzf3	p.R265*	Nonsense
PLKB4.1b3	39397983	13	23647205	C	t		ENSMUSG00000052565	Hist1h1d	p.L83F	Missense
PLKB4.1b3	39398122	13	117814035	A	t		ENSMUSG00000021725	Parp8	p.D52E	Missense
PLKB4.1b3	39397413	14	52740237	C	G		ENSMUSG00000057132	Rpgrip1	p.P182A	Missense
PLKB4.1b3	153044814	15	30548984			GAG	ENSMUSG00000022240	Ctnnd2	p.A98_E99insG	Inframe codon gain
PLKB4.1b3	39393130	15	79725919	G	C		ENSMUSG00000009585	Apobec3	p.L44F	Missense
PLKB4.1b3	39393131	15	79725921	G	T		ENSMUSG00000009585	Apobec3	p.C45F	Missense
PLKB4.1b3	39393141	15	80875717	A	T		ENSMUSG00000042292	Mkl1	c.112+2T>A	Essential splice
PLKB4.1b3	153044906	15	85861442			G	ENSMUSG00000016028	Celsr1	p.S923fs*13	Frameshift insertion
PLKB4.1b3	39393309	15	99611366	C	T		ENSMUSG00000023022	Lima1	p.A382T	Missense
PLKB4.1b3	39393031	15	102260492	A	T		ENSMUSG00000001280	Sp1	p.H648L	Missense
PLKB4.1b3	39393032	15	102260573	A	G		ENSMUSG00000001280	Sp1	p.K675R	Missense
PLKB4.1b3	39393586	16	18252558	G	A		ENSMUSG00000022721	Trmt2a	p.A464T	Missense
PLKB4.1b3	39393632	16	19982838	T	C		ENSMUSG00000043008	Klhl6	p.E71G	Missense
PLKB4.1b3	39393633	16	19982853	C	G		ENSMUSG00000043008	Klhl6	p.C66S	Missense
PLKB4.1b3	39393634	16	19982869	T	G		ENSMUSG00000043008	Klhl6	p.T61P	Missense
PLKB4.1b3	39393493	16	45610678	G	A		ENSMUSG00000022659	Gcet2	c.29+5G>A	Essential splice
PLKB4.1b3	153057770	17	12895130			C	ENSMUSG00000023830	Igf2r	p.D1310fs*5	Frameshift insertion
PLKB4.1b3	39391731	17	29628713	G	C		ENSMUSG00000024014	Pim1	p.S199T	Missense
PLKB4.1b3	39391742	17	31061604	G	A		ENSMUSG00000024027	Glp1r	c.663+5G>A	Essential splice
PLKB4.1b3	39391749	17	31740200	C	T		ENSMUSG00000006705	Pknox1	p.T332M	Missense
PLKB4.1b3	39391769	17	34200375	A	C		ENSMUSG00000024330	Col11a2	p.I1369L	Missense
PLKB4.1b3	39391350	17	35148237	T	G		ENSMUSG00000007029	Vars	p.C478G	Missense
PLKB4.1b3	39391357	17	35332049	A	G		ENSMUSG00000024399	Ltb	p.K73E	Missense
PLKB4.1b3	39391358	17	35332079	A	G		ENSMUSG00000024399	Ltb	p.S83G	Missense
PLKB4.1b3	39391388	17	36462099	C	T		ENSMUSG00000024448	H2-M10.1	p.A174T	Missense
PLKB4.1b3	39391307	17	46967012	A	T		ENSMUSG00000036568	BC032203	c.90+2T>A	Essential splice
PLKB4.1b3	39391308	17	46967043	A	G		ENSMUSG00000036568	BC032203	p.Y21H	Missense

PLKB4.1b3	39391218	17	71346048	A	C		ENSMUSG00000024048	Myl12a	p.L58R	Missense
PLKB4.1b3	39389076	18	50027491	T	G		ENSMUSG00000037416	Dmxl1	p.S801R	Missense
PLKB4.1b3	39389526	19	13009906	T	C		ENSMUSG00000049498	Olfr1449	p.L226P	Missense
PLKB4.1b3	39389428	19	34381769	G	A		ENSMUSG00000024778	Fas	c.184+1G>A	Essential splice
PLKB4.1b3	39397713	X	11616062	T	G		ENSMUSG00000040363	Bcor	p.N1655T	Missense
PLKB4.1b3	39397798	X	109745527	A	C		ENSMUSG00000025529	Zfp711	p.K397Q	Missense
PLKB4.1b3	39397909	X	137077467	C	T		ENSMUSG00000031431	Tsc22d3	p.V14I	Missense
PLKB4.1b4	39354888	1	19094468	C	G		ENSMUSG00000042596	Tfap2d	p.S21R	Missense
PLKB4.1b4	39354787	1	26739735	T	A		ENSMUSG00000073722	4931408C20Rik	p.I1070F	Missense
PLKB4.1b4	39354854	1	58351208	G	A		ENSMUSG00000079554	Aox3l1	p.A247T	Missense
PLKB4.1b4	39354866	1	65306620	G	A		ENSMUSG00000025949	Pikfyve	p.E1574K	Missense
PLKB4.1b4	39354243	1	82285966	C	T		ENSMUSG00000055980	Irs1	p.R368H	Missense
PLKB4.1b4	39354088	1	109503526	T	G		ENSMUSG00000026315	Serpinb8	p.L250R	Missense
PLKB4.1b4	39354128	1	174149628	T	C		ENSMUSG00000007122	Casq1	p.E59G	Missense
PLKB4.1b4	39354710	1	191947259	G	A		ENSMUSG00000010175	Prox1	p.A685V	Missense
PLKB4.1b4	39357681	2	34868531	G	A		ENSMUSG00000026874	Hc	p.A1076V	Missense
PLKB4.1b4	39357684	2	36308487	C	G		ENSMUSG00000058803	Olfr340	p.C127W	Missense
PLKB4.1b4	39357701	2	40716207	C	T		ENSMUSG00000049252	Lrp1b	p.R3100H	Missense
PLKB4.1b4	39358062	2	52578993	T	G		ENSMUSG00000055371	Stam2	p.K48N	Missense
PLKB4.1b4	39358091	2	85681766	A	C		ENSMUSG00000075208	Olfr1019	p.F61V	Missense
PLKB4.1b4	39358097	2	86263012	C	T		ENSMUSG00000078421	Olfr1062	p.A274T	Missense
PLKB4.1b4	39358098	2	86349512	T	G		ENSMUSG00000060742	Olfr1076	p.Y299D	Missense
PLKB4.1b4	39358099	2	86517195	C	G		ENSMUSG00000075175	Olfr1086	p.Q98H	Missense
PLKB4.1b4	39357866	2	88586532	A	G		ENSMUSG00000075117	Olfr1198	p.I171T	Missense
PLKB4.1b4	39357868	2	89246750	G	A		ENSMUSG00000075089	Olfr1238	p.S162F	Missense
PLKB4.1b4	39357812	2	110571699	C	T		ENSMUSG00000050808	Muc15	p.P108S	Missense
PLKB4.1b4	153070869	2	127162366			CTTGCGAGGT	ENSMUSG00000027368	Dusp2	c.393_400+2del<br>CTTGCGAGgt	Complex change in<br>transcript deletion
PLKB4.1b4	153070890	2	136372637			G	ENSMUSG00000074771	Ankrd5	p.G262fs*9	Frameshift deletion
PLKB4.1b4	39357934	2	164181231	G	A		ENSMUSG00000017002	Slpi	p.A68V	Missense
PLKB4.1b4	39358012	2	174253467	A	G		ENSMUSG00000016256	Ctsz	p.M276T	Missense
PLKB4.1b4	39353581	3	38856044	G	C		ENSMUSG00000046743	Fat4	p.V2124L	Missense
PLKB4.1b4	39353594	3	61168181	G	A		ENSMUSG00000053706	B430305J03Rik	p.H22Y	Missense
PLKB4.1b4	39353617	3	72854701	T	C		ENSMUSG00000048304	Slitrk3	p.H220R	Missense
PLKB4.1b4	153062489	3	93250445			GGAGCAAGAG<br>CTCCGCAA	ENSMUSG00000052415	Tchh	p.E626_R627in<br>sQELRKE	Frameshift insertion
PLKB4.1b4	39353758	3	97951233	T	G		ENSMUSG00000027878	Notch2	p.S2403A	Missense
PLKB4.1b4	39355418	4	15831900	G	A		ENSMUSG00000028222	Calb1	p.G259E	Missense
PLKB4.1b4	39355481	4	137834019	C	T		ENSMUSG00000028758	Kif17	p.R327C	Missense
PLKB4.1b4	39357529	6	29559470	G	a		ENSMUSG00000012535	Tnpo3	p.P6S	Missense
PLKB4.1b4	153061070	6	40195653			G	ENSMUSG00000057716	Gm5567	p.G103fs*6	Frameshift deletion
PLKB4.1b4	39357610	6	41721935	T	C		ENSMUSG00000045479	Olfr459	p.D121G	Missense
PLKB4.1b4	39357408	6	55228153	A	G		ENSMUSG00000038022	Fam188b	p.D511G	Missense
PLKB4.1b4	39357416	6	56907279	A	C		ENSMUSG00000071893	Vmn1r4	p.D258A	Missense
PLKB4.1b4	39357460	6	84064057	G	A		ENSMUSG00000033788	Dysf	p.R1060Q	Missense
PLKB4.1b4	39357489	6	124682391	T	C		ENSMUSG00000004266	Ptpn6	p.I85V	Missense
PLKB4.1b4	39357490	6	124682398	C	A		ENSMUSG00000004266	Ptpn6	p.Q82H	Missense
PLKB4.1b4	153061479	6	125121921			C	ENSMUSG00000038252	Ncapd2	p.A976fs*4	Frameshift insertion
PLKB4.1b4	39357372	6	132907367	T	A		ENSMUSG00000057699	Tas2r131	p.K166*	Nonsense
PLKB4.1b4	39358666	7	17740146	G	T		ENSMUSG00000053228	Ceacam3	p.E169*	Nonsense
PLKB4.1b4	39358628	7	25917455	A	C		ENSMUSG00000008496	Pou2f2	p.M6R	Missense
PLKB4.1b4	39358744	7	50915345	C	T		ENSMUSG00000030474	Siglece	p.R53K	Missense
PLKB4.1b4	39358752	7	51784776	C	A		ENSMUSG00000008193	Spib	p.C100F	Missense
PLKB4.1b4	39358753	7	51784853	T	C		ENSMUSG00000008193	Spib	p.N84D	Missense
PLKB4.1b4	39359016	7	70244531	C	T		ENSMUSG00000030525	Chrna7	p.R363H	Missense
PLKB4.1b4	39358860	7	137321263	G	A		ENSMUSG00000030849	Fgfr2	p.Q616*	Nonsense
PLKB4.1b4	39352656	8	12713962	G	A		ENSMUSG00000074469	Gm15348	p.A17V	Missense
PLKB4.1b4	39352665	8	14822586	G	A		ENSMUSG00000047495	Dlgap2	p.R689H	Missense
PLKB4.1b4	39352607	8	28519809	G	A		ENSMUSG00000031491	Chrna6	p.R87C	Missense
PLKB4.1b4	39352608	8	29872206	T	A		ENSMUSG00000063626	Unc5d	p.K237M	Missense
PLKB4.1b4	39352720	8	53282407	A	G		ENSMUSG00000052825	Gm9892	p.S2P	Missense
PLKB4.1b4	39352698	8	88164565	T	C		ENSMUSG00000036902	Neto2	p.Y511C	Missense
PLKB4.1b4	153065841	8	111774830			A	ENSMUSG00000057387	4922502B01Rik	p.L201fs?	Frameshift deletion
PLKB4.1b4	39355541	9	36342022	G	A		ENSMUSG00000074448	Gm5615	p.H73Y	Missense
PLKB4.1b4	39355553	9	38635654	A	G		ENSMUSG00000044798	Olfr923	p.Y126C	Missense
PLKB4.1b4	153066170	9	39509846			G	ENSMUSG00000062121	Olfr149	p.D171fs*2	Frameshift deletion
PLKB4.1b4	39355677	9	53181593	C	T		ENSMUSG00000034584	Exph5	p.T623I	Missense
PLKB4.1b4	39355518	9	73780833	A	C		ENSMUSG00000062151	Unc13c	p.L181R	Missense
PLKB4.1b4	39355898	9	108016984	T	G		ENSMUSG00000032589	Bsn	p.Q1300P	Missense
PLKB4.1b4	39353336	10	23720702	G	T		ENSMUSG00000069705	Taar7b	p.A320S	Missense
PLKB4.1b4	39353340	10	26844287	C	A		ENSMUSG00000019899	Lama2	p.V1682F	Missense
PLKB4.1b4	39353427	10	75296431	C	T		ENSMUSG00000033318	Gstt2	p.V56I	Missense
PLKB4.1b4	39357030	11	48679814	T	G		ENSMUSG00000046879	Irgm1	p.D240A	Missense
PLKB4.1b4	153067348	11	55086649			C	ENSMUSG00000055333	Fat2	p.G3094fs*8	Frameshift deletion
PLKB4.1b4	39356922	11	60310491	G	A		ENSMUSG00000042678	Myo15	p.R763H	Missense
PLKB4.1b4	153067486	11	77728486			G	ENSMUSG00000055045	Gm11190	p.P41fs*23	Frameshift deletion
PLKB4.1b4	39356862	11	90499631	T	G		ENSMUSG00000020544	Cox11	p.L43R	Missense
PLKB4.1b4	39356894	11	98338239	A	T		ENSMUSG00000018168	Ikzf3	c.823+2T>A	Essential splice
PLKB4.1b4	39356895	11	98338271	G	A		ENSMUSG00000018168	Ikzf3	p.R265*	Nonsense
PLKB4.1b4	39354058	12	81211417	C	T		ENSMUSG00000021127	Zfp36l1	p.S59N	Missense
PLKB4.1b4	39353147	13	23647205	C	T		ENSMUSG00000052565	Hist1h1d	p.L83F	Missense
PLKB4.1b4	39353150	13	23647313	C	T		ENSMUSG00000052565	Hist1h1d	p.P119S	Missense
PLKB4.1b4	39352848	14	102039652	A	C		ENSMUSG00000075486	Commd6	p.M1R	Missense
PLKB4.1b4	39356712	15	80875717	A	T		ENSMUSG00000042292	Mkl1	c.112+2T>A	Essential splice
PLKB4.1b4	39356539	15	99611366	C	T		ENSMUSG00000023022	Lima1	p.A382T	Missense
PLKB4.1b4	39356559	15	102260492	A	T		ENSMUSG00000001280	Sp1	p.H648L	Missense
PLKB4.1b4	39356560	15	102260573	A	G		ENSMUSG00000001280	Sp1	p.K675R	Missense
PLKB4.1b4	39354992	16	18252558	G	A		ENSMUSG00000022721	Trmt2a	p.A464T	Missense

PLKB4.1b4	39355040	16	19982838	T	C		ENSMUSG00000043008	Klhl6	p.E71G	Missense
PLKB4.1b4	39355041	16	19982853	C	G		ENSMUSG00000043008	Klhl6	p.C66S	Missense
PLKB4.1b4	39355042	16	19982869	T	G		ENSMUSG00000043008	Klhl6	p.T61P	Missense
PLKB4.1b4	39355078	16	45610678	G	A		ENSMUSG00000022659	Gcet2	c.29+5G>A	Essential splice
PLKB4.1b4	153060833	17	12895130			C	ENSMUSG00000023830	Igf2r	p.D1310fs*5	Frameshift insertion
PLKB4.1b4	39358361	17	29098473	A	C		ENSMUSG00000005936	Kctd20	p.E92A	Missense
PLKB4.1b4	39358382	17	29628477	C	g		ENSMUSG00000024014	Pim1	p.H152D	Missense
PLKB4.1b4	39358391	17	29628981	G	A		ENSMUSG00000024014	Pim1	c.859+5G>A	Essential splice
PLKB4.1b4	39358405	17	31740200	C	T		ENSMUSG00000006705	Pknox1	p.T332M	Missense
PLKB4.1b4	39358166	17	34200375	A	C		ENSMUSG00000024330	Col11a2	p.I1369L	Missense
PLKB4.1b4	39358411	17	35148237	T	G		ENSMUSG00000007029	Vars	p.C478G	Missense
PLKB4.1b4	39358422	17	35332079	A	G		ENSMUSG00000024399	Ltb	p.S83G	Missense
PLKB4.1b4	39358434	17	36462099	C	T		ENSMUSG00000024448	H2-M10.1	p.A174T	Missense
PLKB4.1b4	39358468	17	46967012	A	T		ENSMUSG00000036568	BC032203	c.90+2T>A	Essential splice
PLKB4.1b4	39358469	17	46967043	A	G		ENSMUSG00000036568	BC032203	p.Y21H	Missense
PLKB4.1b4	39358198	17	71346048	A	C		ENSMUSG00000024048	Myl12a	p.L58R	Missense
PLKB4.1b4	39357200	19	13009906	T	C		ENSMUSG00000049498	Olfr1449	p.L226P	Missense
PLKB4.1b4	39357271	19	34381769	G	A		ENSMUSG00000024778	Fas	c.184+1G>A	Essential splice
PLKB4.1b4	153069356	19	53526131			G	ENSMUSG00000025023	5830416P10Rik	p.F108fs?	Frameshift deletion
PLKB4.1b4	39352106	X	11616062	T	G		ENSMUSG00000040363	Bcor	p.N1655T	Missense
PLKB4.1b4	39352171	X	109745527	A	C		ENSMUSG00000025529	Zfp711	p.K397Q	Missense
PLKB4.1b4	39352109	X	153621716	T	C		ENSMUSG00000057457	Phex	p.N633D	Missense

Table S4. Validated mutations from animal PLKE5.1a

Variant ID	Chromosome	Position	WT	Mut	Change	Ensembl gene ID	Gene name	Description	Mutation Consequence
39349870	1	92110418	A	G		ENSMUSG00000044337	Cxcr7	p.Y8C	Missense
153075987	1	120898675			G	ENSMUSG00000048402	Gli2	p.D27fs*15	Frameshift deletion
39350731	2	64771379	A	C		ENSMUSG00000026888	Grb14	p.H236Q	Missense
39350737	2	66073240	T	A		ENSMUSG00000034848	Ttc21b	p.I416L	Missense
39350719	2	87586461	T	C		ENSMUSG00000068817	Olfr1140	p.L36P	Missense
39348525	3	38855777	G	A		ENSMUSG00000046743	Fat4	p.A2035T	Missense
39348654	3	145661547	G	A		ENSMUSG00000036863	Syde2	p.R212H	Missense
39346917	5	20809027	T	A		ENSMUSG00000064280	Ccdc146	p.T614S	Missense
39347126	5	136235040	A	G		ENSMUSG00000019178	Styxl1	p.Y2H	Missense
39346554	6	42306507	A	G		ENSMUSG00000029860	Zyx	p.H456R	Missense
39346461	6	57159903	G	A		ENSMUSG00000064259	Vmn1r13	p.A18T	Missense
39346512	6	77193796	C	T		ENSMUSG00000060780	Lrrtm1	p.R81C	Missense
39346350	6	139991012	T	C		ENSMUSG00000041791	Capza3	p.I272T	Missense
39347964	7	28261330	G	A		ENSMUSG00000055200	Sertad3	p.V57I	Missense
39351106	8	18788375	G	T		ENSMUSG00000039842	Mcph1	p.G771V	Missense
39347512	9	108740842	C	T		ENSMUSG00000023473	Celsr3	p.P2129S	Missense
39347214	9	123765473	A	T		ENSMUSG00000060509	Xcr1	p.F99Y	Missense
39346092	12	47917880	A	C		ENSMUSG00000021047	Nova1	p.F91L	Missense
39346268	12	83442115	A	C		ENSMUSG00000042700	Sipa1l1	p.N43H	Missense
39348937	14	98418095	A	G		ENSMUSG00000055639	Dach1	c.943+2T>C	Essential splice
153073592	16	19953672			C	ENSMUSG00000043008	Klhl6	p.D330fs*19	Frameshift insertion
39347594	16	23113249	A	T		ENSMUSG00000022884	Eif4a2	p.R363S	Missense
39347599	16	30354371	T	A		ENSMUSG00000022533	Atp13a3	p.D258V	Missense
39348310	18	7871607	C	T		ENSMUSG00000024283	Wac	p.R47*	Nonsense
39348486	19	4265302	T	C		ENSMUSG00000034616	Ssh3	p.H332R	Missense
39348462	19	34389717	G	A		ENSMUSG00000024778	Fas	p.C78Y	Missense
39351739	X	17996070	A	G		ENSMUSG00000037358	4930578C19Rik	p.Y424H	Missense
39351859	X	81557853	T	A		ENSMUSG00000045103	Dmd	p.L2267Q	Missense

Table S5. Genotyping primers

cDNA	Forward primer (cDNA)	Reverse primer (tag sequence)
CDK2	GCCTAGCTTTCTGCCATTCTCAT	CATATCGAATACTAGCGGCGC
CDK2.P45L	GCCTAGCTTTCTGCCATTCTCAT	AACGGGCGGTATCTGCTCC
DGKB	TGGAGGTGGTCGGCTTGGAAGGAGC	CTCAGTCCTGCTTAACAAGTGACGCA
DGKB.D592Y	TGGAGGTGGTCGGCTTGGAAGGAGC	CGTTAATCTAGCCGATAAACTTAGTAG
DGKB.G501S	TGGAGGTGGTCGGCTTGGAAGGAGC	GCGGATGCGCAGATCCCGTCGCGGAC
DGKB.K704E	TGGAGGTGGTCGGCTTGGAAGGAGC	ATTAACTCGCCGTGACTGCGTCCAATG
DGKB.M111I	TGGAGGTGGTCGGCTTGGAAGGAGC	CTGCGGAATGCTTATCACGCGGATAAG
DGKB.P432S	TGGAGGTGGTCGGCTTGGAAGGAGC	CGTAGCTGCTATCGCGTACCGAAAAGC
ERN1	CTTCCAGGACGTGAGCGACAG	ATTGATGGCAGGCCGCG
ERN1.S768F	CTTCCAGGACGTGAGCGACAG	TGTCAGGCGTACTAGCCGTCTC
HCK	GTCAAACTTCATGCGGTGGTCA	CGCAATGTACCGAACTAGCACG
HCK.D378G	GTCAAACTTCATGCGGTGGTCA	CGGTAGTGAGCGAATGCTGTC
ITK	AAAGCATTATCACATCAAGGAAACA	GAGGATACGGAACACTTAGCCACTG
ITK.P23L	AAAGCATTATCACATCAAGGAAACA	TTGCGTGGATCTCGATACCTATTC
LYN	GTTTGGGGAAGTCTGGATGG	CATTGTCATCGTGTACGCTCGTC
LYN.D385Y	GTTTGGGGAAGTCTGGATGG	AACCGACTTAGTACCGATTCAATCA
MAPK8	GGCATGGGCTACAAGGAAAAC	GCTCGCTTCATACTGATTCGGTA
MAPK8.G171S	GGCATGGGCTACAAGGAAAAC	CGGGTCTACTGTATAGCGCGTTAG
MAPK8.G177R	GGCATGGGCTACAAGGAAAAC	ATAACCGAAAGTCGCGTAAGCG
MAPK9	CAAAATAAAAACAAGTCAAGCCAGAG	TATCTGTGTACCGCGTAATGGC
MAPK9.K56N	CAAAATAAAAACAAGTCAAGCCAGAG	CGATAGATAGTGCGAAGGATGTGATA
MAPK9.V13M	CAAAATAAAAACAAGTCAAGCCAGAG	ATTATTGTCACTGCGGCGAGG
MGC42105	GAAGCACATGCATGAAAACCAAA	CAGTCTTCGGTATAGTTACGAGTGCA
MGC42105.P411T	GAAGCACATGCATGAAAACCAAA	GCGGGTTTGATTAAGCCTCG
NTRK3	TGAAGCATGGAGACCTGAATAAGT	TTGTCGGTATGCCCTGATCGT
NTRK3.H677Y	TGAAGCATGGAGACCTGAATAAGT	ATTTTCGTTGCTTTCGCGC
NTRK3.R678Q	TGAAGCATGGAGACCTGAATAAGT	GGTCGGCTGCACGCTAAATAG
p53.R270H	CACGTACTCTCCTCCCCTCAATAA	CAAACGATGTCCACCATGATAATATC
YSK4	TCTGGCTATGGACGGAAATCA	AAACCGCATTAGCTCTTATACGAAGT
YSK4.E512V	TCTGGCTATGGACGGAAATCA	TAACTAAACGTGCCGATGGGTAC
